# Vitamin E-Induced Changes in Glutamate and GABA Metabolizing Enzymes of Chick Embryo Cerebrum

**DOI:** 10.1155/2013/851235

**Published:** 2013-07-29

**Authors:** Ivy Pereira, Shanti N. Dessai, Annaliza Pinto

**Affiliations:** ^1^Physiology and Biochemistry Laboratory, Department of Zoology, Goa University, Panaji, Goa 403206, India; ^2^Department of Zoology, Parvatibai Chowgule College of Arts & Science, Gogol, Margao, Goa 403602, India

## Abstract

Vitamin E exists in eight different forms, four tocopherols and four tocotrienols. It forms an important component of our antioxidant system. The structure of Vitamin E makes it unique and indispensable in protecting cell membranes. **α**-tocopherol, one of the forms of Vitamin E, is also known to regulate signal transduction pathways by mechanisms that are independent of its antioxidant properties. Vitamin E compounds reduce the production of inflammatory compounds such as prostaglandins. Swollen, dystrophic axons are considered as the hallmark of Vitamin E deficiency in the brains of rats, monkeys, and humans. The present work aimed to study the Vitamin E- (**α**-tochopherol acetate-) induced alterations of enzymes involved in metabolism of Glutamate and GABA during developmental neurogenesis of cerebrum. Therefore, cytosolic and crude mitochondrial enzyme activities of glutamine synthetase, aspartate transaminase, alanine transaminase, GABA transaminase, succinic Semialdehyde dehydrogenase, glutamic dehydrogenase, and **α**-Ketoglutarate dehydrogenase were analysed. Vitamin E induced significant changes in these enzymes thus altering the normal levels of glutamate and GABA during developmental neurogenesis. Such changes are surely to disturb the expression and/or intensity of neurotransmitter signaling during critical periods of brain development.

## 1. Introduction

Vitamin E activity was first identified in the year 1936 from a dietary fertility factor in rats. Tocopherols (or TCPs) constitute a class of chemical compounds which possess Vitamin E activity. They are series of organic compounds consisting of various methylated phenols. Vitamin E exists in eight different forms, four tocopherols and four tocotrienols. Each form has a chromanol ring, with a hydroxyl group that can donate a hydrogen atom to reduce free radicals and a hydrophobic side chain which allows for penetration into biological membranes. Both the tocopherols and tocotrienols occur in alpha, beta, gamma, and delta forms, designated by the number of methyl groups on the chromanol ring. The alpha is most highly methylated having maximum of three methyl groups on the chromanol ring in comparison with beta, gamma, and delta forms having two, one, and no methyl groups on the chromanol ring, respectively. Synthetic Vitamin E derived from petroleum products is manufactured as all-racemic alpha tocopheryl acetate with a mixture of eight stereoisomers. In this mixture, one alpha-tocopherol molecule in eight molecules is in the form of *RRR*-alpha-tocopherol. The eight stereoisomers of alpha-tocopherol differ in the arrangement of groups around these stereocenters. 1 IU is defined as 1 milligram of an equal mix of the eight stereoisomers, which is a racemic mixture called *all-rac*-alpha-tocopheryl acetate. This mixture of stereoisomers is often called dl-alpha-tocopheryl acetate, even though it is more precisely dl, dl, dl-alpha-tocopheryl acetate [1]. 

Vitamin E is known to influence a wide range of mechanisms underlying human health and disease [2]. Its importance for normal neurological function [3, 4] is well designated by research based on Vitamin E deficiency studies which demonstrated its central role in maintaining neurological structure and function [4]. Neuroprotective effects of Vitamin E are due to its antioxidant property. Vitamin E-deficient diets lead to encephalomalacia and ataxia in growing chicks, ataxia, and neuroaxonal degeneration in brain stem, spinal cord, and peripheral nerves and pigmentary degeneration of retina develops in rats and rhesus monkeys [5–8]. Swollen, dystrophic axons, the hallmark of Vitamin E deficiency, have been also observed in the brains of rats, monkeys, and humans [9]. Vast literature available on Vitamin E studies hardly emphasizes its role during prenatal development. Despite of the fact that Vitamin E activity was first documented on dietary fertility factor in rats there are very few reports which actually emphasize its role during embryonic/prenatal development. It is also well known to be protective against glutamate-induced neurotoxicity in adults but remains deficient to focus its actions against high glutamate and other free amino acid pool during developmental neurogenesis where these amino acids serve as neuroregulators and modulators for neurogenesis. 

The objective of this study was to highlight the potential effects of the *α*-tochopherol acetate during developmental neurogenesis with special reference to enzymes involved in the metabolism of glutamate and GABA of developing cerebrum. With an intention to avoid mystifying factors of maternal/placental drug effects, nonplacental chick embryo model was selected for present study [10].

## 2. Materials and Methods

### 2.1. Experimental Setup

Fertilized eggs (white Leghorn strain) obtained from local hatchery of Goa Institute of Rural Development and Administration, Ela Farm, Old Goa, Goa, India. After being brought to laboratory they were cleaned of any external dirt with distilled water and then with 70% alcohol. They were candled before incubation to remove chapped and broken eggs. Nonchapped and nonbroken fertilized eggs were selected and weighed. Eggs in weight range of 45–50 g were selected for experimentation. Selected eggs were then segregated in four groups of twenty eggs in each (Groups I, II, III, and IV) and were transferred to humidified incubator set at 37.8°C temperature. This day was marked as day 0. Necessary periodic rotations of the eggs were done during first 2 days of incubation. 

### 2.2. *In Ovo* Exposure

On the third day, that is, at the end of 72 hrs of incubation all the eggs were taken out of incubator and wiped with 70% alcohol and transferred to laminar flow hood. At a point about one-half inch from the small end of the egg, a small hole in the shell with a dissecting needle was made on egg shell of Groups II, III, and IV eggs. The hole was just large enough for the hypodermic needle to go in. Yolk sac injections were performed through this hole within the egg and syringe in horizontal position. All operations were performed in a laminar hood to insure sterility. The selection of these *α*-tochopherol acetate doses was based on the research data reported by previous investigators [11–13]. Group I: intact control set which did not receive any vehicle (oil) and test compound (*α*-tochopherol acetate); Group II: vehicle Control set which received 0.1 mL of oil (vehicle); Group III: experimental set which received 0.1 mL of *α*-tochopherol acetate dose of 75 *μ*g/g of yolk. Group IV: experimental set which received 0.1 mL of *α*-tochopherol acetate dose of 150 *μ*g/g of yolk. After each injection, a hole on each egg was closed with molten wax. All sets were then transferred back to humidified incubator set at 37.8°C temperature for further incubation. On day 15 ten eggs from each set were sacrificed and remaining ten eggs were sacrificed after completion of 20 days of incubation.

### 2.3. Enzyme Sample Preparation

On day 15 (ten eggs from each group) and 20 (remaining ten eggs from each group) of incubation embryos were sacrificed by decapitation without anesthesia. Brains were rapidly excised on a petridish placed on ice. The brain regions other than cerebral hemisphere were discarded. Cerebral hemispheres were dissected and kept chilled until homogenization with a ground glass type Potter-Elvehjem homogenizer. Cerebral hemispheres from two brains were pooled to provide one sample; thus five samplings were done for each group of 15 day and 20 day incubated sets. For cytosolic enzyme assay, 10% homogenate was prepared in ice-cold SH buffer containing 0.32 M sucrose and 5 mM Hepes (pH 7.4). Homogenates were then centrifuged at 4°C at 1000 ×g for 10 min [14]. The supernatant thus received was collected and was used as cytosolic enzyme source for enzyme assays. For mitochondrial enzyme assay, 10% homogenate was prepared in ice-cold SH buffer containing 0.32 M sucrose and 5 mM Hepes (pH 7.4). Homogenates were then centrifuged at 4°C at 1000 ×g for 10 min [14]. The supernatant thus received was further centrifuged further at 4°C at 10,700 ×g for 20 min [14]. Crude mitochondrial pellet received at the end of centrifugation was collected and was then diluted with same buffer which served as an enzyme source for crude mitochondrial fraction.

### 2.4. Enzyme Assays

All the enzyme assays were carried out using UV-Vis Double beam Spectrophotometer (Schimadzu, UV-2450 UV visible spectrophotometer). Absorbance of reaction mixtures of protein, aspartate, and alanine aminotransferases were read on Photometric Mode whereas absorbance of reaction mixtures of rest all cuvette assays with continuous spectrophotometric rate determination were read on Time Kinetic Mode of the previously mentioned spectrophotometer.

### 2.5. Glutamine Synthetase (GS) (EC 6.3.1.2)

GS activity was determined by following the method of Rowe [15]. 3.00 mL final assay reaction mixture contained 34.1 mM imidazole, 102 mM sodium glutamate, 8.5 mM adenosine 5′-triphosphate, 1.1 mM phosphoenolpyruvate, 60 mM magnesium chloride, 18.9 mM potassium chloride, 45 mM ammonium chloride, 0.25 mM *β*-nicotinamide adenine dinucleotide, 28 units pyruvate Kinase, and 40 units L-lactic dehydrogenase with 0.1 mL of enzyme source. One unit of enzyme activity was designated to amount of enzyme activity that converts 1.0 *μ*mole of L-glutamate to L-glutamine in 15 minutes at pH 7.1 at 37°C.

### 2.6. Aspartate Transaminase (AAT) (EC 2.6.1.1)

AAT was measured according to 2,4-dinitrophenyl hydrazine (2,4-DNPH) method. The incubation mixture for AAT contained 500 *μ*L of aspartate-*α*-ketoglutarate, 100 *μ*L of enzyme extract, 500 *μ*L of 2,4-DNPH, and 5 mL of 0.4 N NaOH. Optical density of corresponding brown coloured hydrazone formed in alkaline medium was read at 505 nm. One unit of enzyme (U/mg protein) was defined as 1 mmol of pyruvate liberated per minute at 37°C incubation per mg protein. 

### 2.7. Alanine Aminotransferase (ALAT) (E.C. 2.6.1.2)

ALAT was measured according to 2,4-dinitrophenyl hydrazine (2,4-DNPH) method. The incubation mixture for ALAT contained 500 *μ*L of alanine-*α*-ketoglutarate, 100 *μ*L of enzyme extract, 500 *μ*L of 2,4-DNPH, and 5 mL of 0.4 N NaOH. Optical density of corresponding brown colored hydrazone formed in alkaline medium was read at 505 nm. One unit of enzyme (U/mg protein) was defined as 1 mmol of pyruvate liberated per minute at 37°C incubation per mg protein. 

### 2.8. GABA Transaminase (GABA-T) (EC 2.6.1.19)

GABA-T activity was determined by following the method of Sherif et al. [16]. 3.02 mL final assay reaction mixture contained 86 mM potassium pyrophosphate, 3.3 mM 2-mercaptoethanol, 1.2 mM *β*-nicotinamide adenine dinucleotide phosphate, 5 mM a-ketoglutarate, 6.0 mM *γ*-amino-n-butyric acid, 0.50 mM potassium phosphate, and 0.17% (v/v) glycerol with 0.02 mL of enzyme source. One unit of enzyme activity was designated to amount of enzyme activity that converts 1.0 *μ*mole of *γ*-aminobutyric acid (GABA) to succinic semialdehyde and then to succinate per minute with a stoichiometric reduction of 1.0 *μ*mole of NADP^+^ at pH 8.6 at 25°C.

### 2.9. Succinic Semialdehyde Dehydrogenase (SSADH) (EC 1.2.1.16)

SSADH activity was determined by following the method of Ryzlak and Pietruszko [17]. 3.00 mL final assay reaction mixture contained 87 mM potassium pyrophosphate, 3 mM 2-mercaptoethanol, 1.3 mM *β*-nicotinamide adenine dinucleotide phosphate, 5.0 mM succinic semialdehyde, 0.83% (v/v) glycerol, and 2.5 mM potassium phosphate with 0.1 mL of enzyme source. One unit of enzyme activity was designated to amount of enzyme activity that converts 1.0 *μ*mole of succinic Semialdehyde to succinate per minute with a stoichiometric reduction of 1.0 *μ*mole of NADP^+^ at pH 8.6 at 25°C.

### 2.10. L-Glutamic Dehydrogenase (GDHH) (EC 1.4.1.3)

GDH activity was determined by following the method of Fisher [18]. 3.00 mL final assay reaction mixture contained 90 mM triethanolamine hydrochloride, 13 mM *α*-ketoglutarate, 53 mM ammonium acetate, 0.06 mM *β*-NADH, and 0.25 mM EDTA with 0.1 mL of enzyme source. One unit of enzyme activity was designated to amount of enzyme activity that reduces 1.0 *μ*mole of *α*-ketoglutarate to L-glutamate per minute at pH 7.3 at 25°C, in the presence of ammonium ions.

### 2.11. *α*-Ketoglutarate Dehydrogenase (*α*-KGDHH) (EC 1.2.4.2)

Reaction mixture for A-KGDHH activity contained 50.8 mM MOPS, 0.2 mM MgCl_2_, 0.01 mM CaCl_2_, 0.3 mM cocarboxylase, 0.12 mM coenzyme A, 2.0 mM *β*-nicotinamide adenine dinucleotide, 2.6 mM L-cysteine, and 5.0 mM *α*-ketoglutaric acid with 0.05 mL of enzyme source. One unit of enzyme activity was designated to amount of enzyme activity that converts 1.0 *μ*mole of *β*-NAD to *β*-NADH per minute at pH 7.4 at 30°C in the presence of saturating levels of coenzyme A.

### 2.12. Protein Assay

Protein concentration was measured according to Lowry et al. [19] using bovine serum albumin as the standard.

### 2.13. Statistical Analysis

All values represented in tables and figures are expressed as means of five-sampling ± standard deviation. Statistical significance of differences were determined by using unpaired Student's *t*-test (WINOWS 7 EXCEL) to compare control (Group I) against experimental groups (Groups II, III, and IV). *P* < 0.05 was considered statistically significant. Two-way anova was done with the help of Analyse-it Program for General and Clinical statistics version 1.73. Variations in the each enzyme activity under the influence of Vitamin E (Groups I, II, III, and IV) for two incubation periods (15 days and 20 days) were represented as *F* (*F* ratio) with “degree of freedom”, df = 3,1.

## 3. Results

### 3.1. Glutamine Synthetase

**Figure 1:**
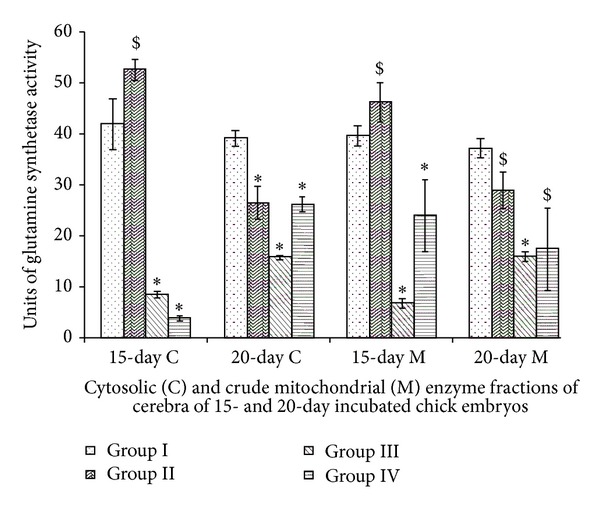
Vitamin E-induced changes of glutamine synthetase activity of cerebrum of chick embryo. Data are mean ± SD for five independent experiments performed. *Different from control, *P* < 0.001; ^$^ *P* < 0.01 (Student's *t*-test). Group I: intact control; Group II: vehicle control; Group III received 0.1 mL of *α*-tochopherol acetate dose of 75 *μ*g/g of yolk and Group IV received 0.1 mL of *α*-tochopherol acetate dose of 150 *μ*g/g of yolk.

Vitamin E-treated chick embryo cerebrum showed decrease in GS activity in comparison to control Group I chick embryo cerebrum. Whereas oil-treated Group II cerebra showed increase in cytosolic and mitochondrial activity of 15-day incubated chick embryos. Major decline of GS activity in all Vitamin E-treated groups in comparison to Group I (Figure 1). Two-way ANOVA between all groups (Group I, II, III, and IV) and incubation periods (15- and 20-days incubated chick embryos) for cytosolic (df = 3,1; *F* = 186.39) and mitochondrial (df = 3,1; *F* = 16.198) GS activities were significant at *P* < 0.0001.

### 3.2. Aspartate Aminotransferase

**Figure 2:**
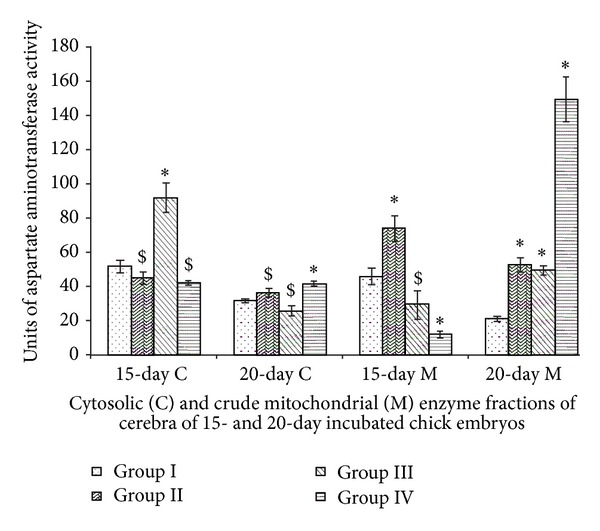
Vitamin E-induced changes of aspartate aminotransferase activity of cerebrum of chick embryo. Data are mean ± SD for five independent experiments performed. *Different from control, *P* < 0.001; ^$^ *P* < 0.01 (Student's *t*-test). Group I: intact control; Group II: vehicle control; Group III received 0.1 mL of *α*-tochopherol acetate dose of 75 *μ*g/g of yolk and Group IV received 0.1 mL of *α*-tochopherol acetate dose of 150 *μ*g/g of yolk.

Cytosolic AAT activity of 15-day incubated chick embryo of Group III showed sharp increase in activity whereas other group enzyme activities did not show any major changes in comparison to Group I. So also 20-day incubated chick embryo cytosolic AAT activity did not show much outstanding changes in their activities. Mitochondrial AAT activity of 15-day incubated chick embryo cerebra of Group II showed increase while other groups such as Groups III and IV marked decline in their activities. However, Mitochondrial activity of all groups of 20-day incubated chick embryos showed elevations. Maximum spike in mitochondrial AAT activities of Group IV of 20-day incubated chick embryo was very conspicuous (Figure 2). Two-way ANOVA between all groups (Groups I, II, III, and IV) and incubation periods (15- and 20-day incubated chick embryos) for cytosolic (df = 3,1; *F* = 148.29) and mitochondrial (df = 3,1; *F* = 335.60) AAT activities were significant at *P* < 0.0001.

### 3.3. Alanine Aminotransferases

**Figure 3:**
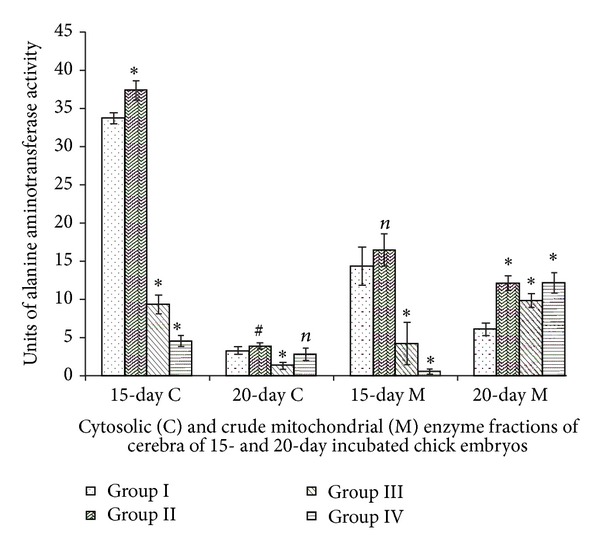
Vitamin E-induced changes of alanine aminotransferase activity of cerebrum of chick embryo. Data are mean ± SD for five independent experiments performed. *Different from control, *P* < 0.001; ^#^ *P* < 0.05; *n*: insignificant (Student's *t*-test). Group I: intact control; Group II: vehicle control; Group III received 0.1 mL of *α*-tochopherol acetate dose of 75 *μ*g/g of yolk and Group IV received 0.1 mL of *α*-tochopherol acetate dose of 150 *μ*g/g of yolk.

Cytosolic and mitochondrial activities of Group II showed minor increments. Major decline in cytosolic and mitochondrial enzyme activities was seen in Groups III and IV of 15-day incubated chick embryos. Cytosolic ALAT activities of 20-day incubated chick embryo cerebra showed minor decline in Group III in comparison to Group I control. Major declines of cytosolic ALAT activities of 15-day incubated chick embryo cerebra of Groups III and IV under the influence of Vitamin E were well marked. Whereas, 20-day incubated chick embryo cerebra of Group II, III, and IV showed elevations (Figure 3). Two-way ANOVA between all groups (Group I, II, III and IV) and incubation periods (15- and 20-days incubated chick embryos) for cytosolic (df = 3,1; *F* = 965.29) and mitochondrial (df = 3,1; *F* = 64.36) ALAT activities were significant at *P* < 0.0001.

### 3.4. GABA Transaminases

**Figure 4:**
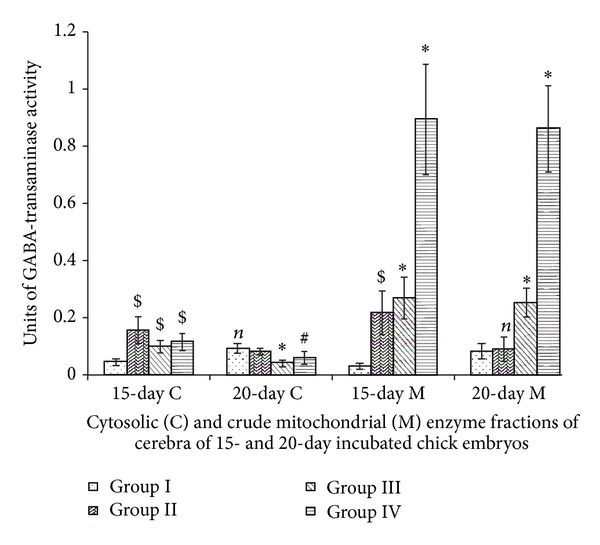
Vitamin E-induced changes of GABA-transaminase activity of cerebrum of chick embryo. Data are mean ± SD for five independent experiments performed. *Different from control, *P* < 0.001; ^$^ *P* < 0.01; ^#^ *P* < 0.05; *n*: insignificant (Student's *t*-test). Group I: intact control; Group II: vehicle control; Group III received 0.1 mL of *α*-tochopherol acetate dose of 75 *μ*g/g of yolk and Group IV received 0.1 mL of *α*-tochopherol acetate dose of 150 *μ*g/g of yolk.

Cerebral cytosolic and mitochondrial GABA-T activities of Group II, 15-day incubated chick embryos showed elevations in comparison to Group I. Vitamin E induced increments in mitochondrial enzyme activities of cerebra of 15-day incubated chick embryo and also increased mitochondrial enzyme activities of cerebra of 15-day and 20-day incubated chick embryos of III and IV Groups in comparison to Group I. Maximum spikes of mitochondrial enzyme activity were observed for Group IV (Figure 4). Two-way ANOVA between all groups (Groups I, II, III, and IV) and incubation periods (15- and 20-day incubated chick embryos) for cytosolic (df = 3,1; *F* = 12.859) and mitochondrial (df = 3,1; *F* = 64.360) GABA-T activities were significant at *P* < 0.0001.

### 3.5. Succinic Semialdehyde Dehydrogenase

**Figure 5:**
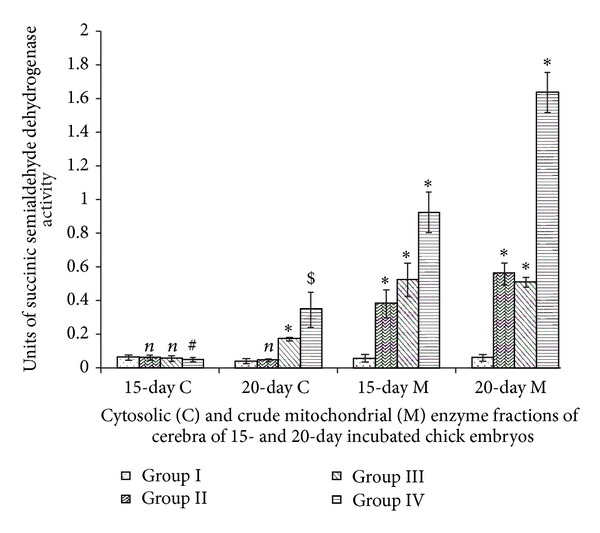
Vitamin E-induced changes of succinic semialdehyde dehydrogenase activity of cerebrum of chick embryo. Data are mean ± SD for five independent experiments performed. *Different from control, *P* < 0.001; ^$^ *P* < 0.01; ^#^ *P* < 0.05; *n*: insignificant (Student's *t*-test). Group I: intact control; Group II: vehicle control; Group III received 0.1 mL of *α*-tochopherol acetate dose of 75 *μ*g/g of yolk and Group IV received 0.1 mL of *α*-tochopherol acetate dose of 150 *μ*g/g of yolk.

Group II showed elevated mitochondrial SSADH activity in comparison to Group I. Vitamin E induced elevations of cytosolic (20-day incubated chick embryos only) and mitochondrial (15-day and 20-day incubated chick embryos) enzyme activities (Figure 5). Two-way ANOVA between all groups (Groups I, II, III, and IV) and incubation periods (15- and 20-day incubated chick embryos) for cytosolic (df = 3,1; *F* = 42.123) and mitochondrial (df = 3,1; *F* = 45.711) SSADH activities were significant at *P* < 0.0001.

### 3.6. Glutamine Dehydrogenase

**Figure 6:**
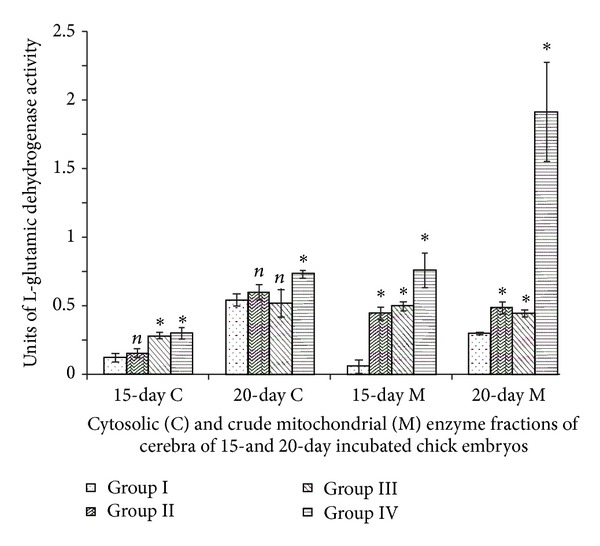
Vitamin E-induced changes of L-glutamic dehydrogenase activity of cerebrum of chick embryo. Data are mean ± SD for five independent experiments performed. *Different from control, *P* < 0.001; *n*: insignificant (Student's *t*-test). Group I: intact control; Group II: vehicle control; Group III received 0.1 mL of *α*-tochopherol acetate dose of 75 *μ*g/g of yolk and Group IV received 0.1 mL of *α*-tochopherol acetate dose of 150 *μ*g/g of yolk.

Cerebral mitochondrial GDH activities of Groups II, III, and IV of 15-day incubated chick embryo showed great increments compared to Group I. Mitochondrial activity of Group IV showed conspicuous elevations (Figure 6). Two-way ANOVA between all groups (Groups I, II, III, and IV) and incubation periods (15- and 20-day incubated chick embryos) for cytosolic (df = 3,1; *F* = 8.876) and mitochondrial (df = 3,1; *F* = 39.570) GDH activities were significant at *P* < 0.001 and *P* < 0.0001, respectively.

### 3.7. *α*-Ketoglutarate Dehydrogenase

**Figure 7:**
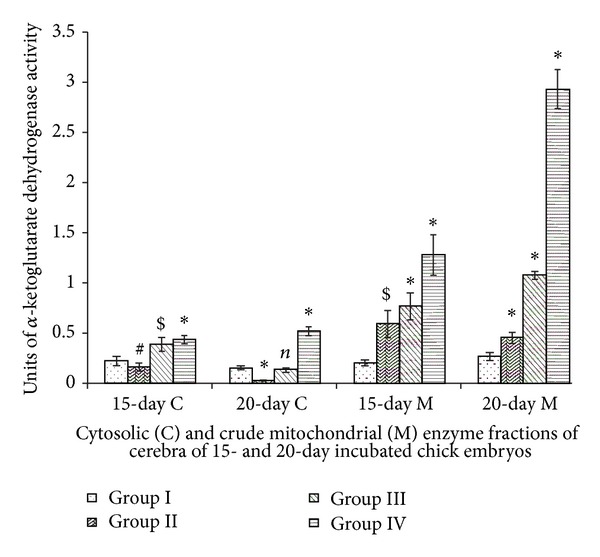
Vitamin E-induced changes of *α*-ketoglutarate dehydrogenaseactivity of cerebrum of chick embryo. Data are mean ± SD for five independent experiments performed. *Different from control, *P* < 0.001; ^$^ *P* < 0.01; ^#^ *P* < 0.05; *n*: insignificant (Student's *t*-test). Group I: intact control; Group II: vehicle control; Group III received 0.1 mL of *α*-tochopherol acetate dose of 75 *μ*g/g of yolk and Group IV received 0.1 mL of *α*-tochopherol acetate dose of 150 *μ*g/g of yolk.

Cerebral cytosolic A-KGDHH activities of Group III (15-day incubated embryos) and Group IV (15-day and 20-day incubated embryos) showed elevations. Prominent elevations were observed in mitochondrial enzyme activities. The is outstanding rise in mitochondrial activities of Group IV under the influence of Vitamin E (Figure 7). Two-way ANOVA between all groups (Groups I, II, III, and IV) and incubation periods (15- and 20-day incubated chick embryos) for cytosolic (df = 3,1; *F* = 32.621) and mitochondrial (df = 3,1; *F* = 107.313) A-KGDHH activities were significant at *P* < 0.0001.

## 4. Discussion 

Based on variety of studies, the developing central nervous system appears to be especially sensitive to adverse effects [10]. Embryo developing *in ovo* seems to be more susceptible to oxidative damage due to its high content of unsaturated fatty acids and relative embryonic deficiencies in free radical-related cytoprotective enzymes [20]. 

GS is one of the key enzymes of brain because of its involvement in the synthesis of nucleic acids and mucopolysaccharides and in the metabolism of neurotransmitters [21]. GS catalyzes the conversion of ammonia and glutamate to glutamine. Vertebrate nervous system shows presence of GS throughout the brain and serves to detoxify the brain ammonia and also helps in metabolic regulation of glutamate. Its activity is developmentally regulated [22, 23] and shows regional variations in the distribution and developmental pattern of GS activity in brain. The increase in GS activity is associated with astrocyte differentiation rather than proliferation. It is also known fact that distribution of GS is also dependent on local levels of extracellular glutamate. One of the most important roles of astrocytes in brain is to protect neurons against excitotoxicity by capturing excess ammonia and glutamate and converting it into glutamine via the enzyme GS. Glutamine thus released to the extracellular space is made available to the neurons as a glutamate precursor. Glutamate released by neurons can then be captured by astrocytes and reconverted into glutamine. Normal levels of ammonia in brain appear to lead to rapid astrocytic conversion of glutamate to glutamine, thus keeping intracellular glutamate at a very low level. Thus, GS activity influences not only the brain's capacity to remove ammonia but also glutamate flux. In addition, it has been suggested that activation of GS may provide astrocytic protection against hypoxic injury [24]. Present study showed Vitamin E-induced inactivation of glutamine synthetase in chick embryo cerebrum, thus affecting synthesis of nucleic acids and mucopolysaccharides and metabolism of neurotransmitters. Such inactivation would thus bring about changes in differentiating pattern of astrocytes in developing brain of chick embryo [24].

The AAT has been implicated to be of key importance to both the synthesis of glutamate from 2-oxoglutarate and regulating the relative glutamate level in the presynaptic terminal. AAT is one of the most active enzymes in brain comparable in activity to those enzymes associated with the glycolysis or cell respiration. The most rapid changes in enzyme activities occur during the second phase of maturation referred to as the “critical phase” of morphological and functional development of the nervous system which are marked phases of late embryonic development and postnatal development [25]. Glutamate, an important excitatory neurotransmitter, activates both ionotropic (ligand-gated ion channels) and metabotropic (G protein-coupled) receptors in developing brain. Abundance and widespread distribution of glutamate in embryonic life is also a known fact. Glutamate also acts to influence earlier developmental events, some of which occur prior to synapse formation, such as proliferation, migration, differentiation, or survival processes during neural development. To fulfill these actions in the constructing of the nervous system, different types of glutamate receptors need to be expressed both at the right time and at the right place. Proliferation of neuronal progenitors is one of the fundamental developmental processes responsible for generating the correct number of cells of each type in the correct sequence in the brain. Cell-intrinsic as well as cell-extrinsic factors contribute to changes in cell production thus affecting the cerebral cortical growth. Among other extracellular molecules, neurotransmitter receptors have been implicated in the extrinsic regulation of cell proliferation in the developing brain. Neurotransmitter receptors are also involved in the proliferation and migration of cortical neurons as well [26]. Therefore, Vitamin E-induced elevations of cerebral AAT activity suggest stepped up synthesis of glutamate which may affect these proliferative changes in the chick embryo cerebrum.

ALAT occupies an important position between pyruvate and glutamate metabolism. It is also known to control pyruvate, glutamate, and ammonia metabolism in brain [27]. Compared to other brain regions its levels are higher in cortical gray matter [28]. Vitamin E-induced inactivation of ALAT observed in present study would lead to disturbances in the linkages between carbohydrate and protein metabolism, causing energy crisis as well as glutamate metabolism during development.

GABA-T helps in metabolic removal GABA. The fraction of GABA taken up by neurons is mainly recycled into synaptic vesicles and is used repeatedly for synaptic communication. In astrocytes, a large part of GABA is metabolized via the citrate cycle. Through this pathway, GABA can serve as an intermediary metabolic compound. GABA is transformed to succinic semialdehyde by transamination reaction catalyzed by the GABA-T enzyme. GABA-T is associated with the inner mitochondrial membrane and also seen sometimes in the cytoplasm and its activity plays an important role in setting the GABA level of the local tissue environment. Elevation of GABA-T activity provides a possible tool for setting extracellular GABA concentration in various circumstances [29]. Vitamine E-induced elevations of GABA-T suggest that there is high amount of GABA for its metabolic removal. Literature also demonstrates that GABA signalling possess an inherent capability for potent regulation of almost all steps of neuronal differentiation and neural tissue formation. Differentiating neuronal populations show transient GABA production, regardless of the future neurotransmitter phenotype. In the developing nervous tissue, GABA acts as an autocrine/paracrine signal molecule and its main roles are executed through tonic signaling. In young differentiating neurons, most important effects of GABA are mediated through GABAA receptors and result in membrane depolarization and in an increased [Ca^+2^]_I_ which in turn can stimulate multiple cellular processes. GABAB receptor-mediated effects, which can cause hyperpolarization and reduce [Ca^+2^]_I_, appear in later phases of tissue genesis and seem to balance GABA signaling. The effects of GABA had been demonstrated in the entire period of neural tissue genesis, from proliferation, through migration and differentiation of neuronal precursors, up to the synapse formation and circuit refinement by maturing neurons. The most intriguing developmental role that has been attributed to GABA is the generation and maintenance of activity waves in the period of functional network formation [29]. Elevated levels of GABA-T under the influence of Vitamin E indicate presence of high amounts of GABA available for its metabolic removal. Therefore, elevation of GABA-T encountered in the present study may be referred to as compensatory elevations to remove these high amounts of GABA which otherwise may interfere with the normal neurogenesis in cerebrum leading to overactivation of proliferative and differentiative events.

SSADH brings about oxidation of succinic semialdehyde to produces succinate which enters the Krebs cycle of brain cell. This shows its importance in GABA degradation [30]. Therefore, Vitamin E-induced elevations of SSADH in present study indicate its elevated role towards the degradation of very high levels of GABA in developing cerebral tissue. High levels of GABA also suggest high levels of glutamate available for its conversion to GABA that would otherwise interfere with normal neurogenesis pathways.

GDH is one of the main enzymes involved in the formation and metabolism of the neurotransmitter glutamate and is well marked in cerebrum, a region rich in glutamatergic neurons. GDH catalyses reversible oxidative deamination of L-glutamate to *α*-oxoglutarate using NAD/NADP as coenzymes. According to most biochemical studies, GDH is ubiquitously detected in neurons and glia [31]. The peculiar position of GDH in the intermediary metabolism as an interface between the two oxidoreductive systems of NAD and NADP-linked enzymes, between carbohydrates and amino acids and in the vicinity of the citric acid cycle and the urea cycle, offers various possibilities for the physiological functions of the enzyme. A role of GDH in the generation of ammonia for the urea cycle is implied by the fact that deamination of glutamate yields ammonia. The traditional view is that this molecule is incorporated into carbamyl phosphate which enters the urea cycle [32]. Such elevations are known to correlate with synaptogenesis of mainly parallel fibres [33]. Increased GDH activity also indicates a functional significance of this enzyme in regions which contain glutamatergic terminals, being associated with the small glutamate pool. The rapid rise of this enzyme activity in developing brain regions might be attributed to the glutamatergic terminals, that is, to the small glutamate pool which, in turn, is connected with the development of glutamatergic transmission processes [34].

The A-KGDHH is a control point of the tricarboxylic acid cycle in tissues. The overall transformation catalyzed by *α*-ketoglutarate dehydrogenase involves the sequential actions of *α*-ketoglutarate dehydrogenase, dihydrolipoamide succinyl transferase and dihydrolipoamide dehydrogenase [35]. *α*-ketoglutarate is crucial in the cellular production of reducing equivalents (NADH) and in the maintenance of the mitochondrial redox state of the brain. The mitochondrial membrane potential, a reaction of this redox state, is linked to numerous aspects of brain biochemistry including neurotransmitter release. *α*-ketoglutarate reaction is central for the catabolism of the carbon skeleton of glutamate, which is important as a neurotransmitter and for protein synthesis [36]. In the present study, elevated A-KGDHH activity under the influence of Vitamin E suggests elevated availability of *α*-ketoglutarate that needs to be converted to succinyl CoA for its further metabolic removal as succinate.

## 5. Conclusion

Vitamin E-induced considerable changes in the previously discussed enzymes involved metabolism of glutamate and GABA. Normally, levels of these two amino acids are maintained high during developmental neurogenesis due to their role in proliferation and differentiation of various neuronal cell types which seems to be in agreement with the present study. Based on these enzyme studies, it can be inferred that levels of glutamate and GABA were definitely increased under the influence of Vitamin E in Groups III and IV much above the levels of Glutamate and GABA as normally seen in developing brain. Such extraordinary increments are likely to disturb the timetable of expression and/or intensity of neurotransmitter signaling during critical periods of brain development which may lead to permanent changes in the formation of neuronal circuits by affecting neurogenesis, neuronal migration, neurite arborisation, and establishment of synaptic connections. This study also focused on the fact that enzyme pathways leading to conversion of glutamate and GABA to succinate were activated thus showing some kind of compensatory conversion of glutamate and GABA to succinate which do not participate in developmental neurogenesis.
